# Perceptions and use of the national kidney foundation KDOQI guidelines: a survey of U.S. renal healthcare providers

**DOI:** 10.1186/1471-2369-14-230

**Published:** 2013-10-24

**Authors:** Michelle M Estrella, Bernard G Jaar, Kerri L Cavanaugh, Chester H Fox, Mark A Perazella, Sandeep S Soman, Emily Howell, Michael V Rocco, Michael J Choi

**Affiliations:** 1 Departments of Medicine, Johns Hopkins University School of Medicine, 1830 E. Monument Street, Suite 416, Baltimore, MD 21205, USA; 2 Department of Epidemiology, Johns Hopkins University Bloomberg School of Public Health, 1830 E. 615 North Wolfe Street, Baltimore, MD 21205, USA; 3 Department of Medicine, Nephrology Center of Maryland, 5601 Loch Raven Boulevard, Suite 3 North, Baltimore, MD 21239, USA; 4 Department of Medicine, Vanderbilt University Medical Center, 1161 21stAvenue, SouthMedical Center North S-3223, Nashville, TN 37232, USA; 5 Department of Family Medicine, University at Buffalo School of Medicine and Biomedical Sciences, 1315 Jefferson Avenue, Buffalo, NY 14208, USA; 6 Department of Medicine, Yale School of Medicine, 2799 West Grand Boulevard, P.O. Box 208029, New Haven, CT 06520-8029, USA; 7 Department of Internal Medicine, Henry Ford Hospital, 2799 West Grand Boulevard, Detroit, MI 48202, USA; 8 National Kidney Foundation, 30 East 33rd Street, New York, NY 10016, USA; 9 Department of Medicine, Wake Forest University School of Medicine, Medical Center Boulevard, Winston-Salem, NC 27157-1053, USA

**Keywords:** KDOQI, Chronic kidney disease, Guidelines, Survey

## Abstract

**Background:**

The National Kidney Foundation (NKF) Kidney Disease Outcomes Quality Initiative (KDOQI) developed guidelines to care for patients with chronic kidney disease (CKD). While these are disseminated through the NKF’s website and publications, the guidelines’ usage remains suboptimal. The KDOQI Educational Committee was formed to identify barriers to guideline implementation, determine provider and patient educational needs and develop tools to improve care of patients with CKD.

**Methods:**

An online survey was conducted from May to September 2010 to evaluate renal providers’ familiarity, current use of and attitudes toward the guidelines and tools to implement the guidelines.

**Results:**

Most responders reported using the guidelines often and felt that they could be easily implemented into clinical practice; however, approximately one-half identified at least one barrier. Physicians and physician extenders most commonly cited the lack of evidence supporting KDOQI guidelines while allied health professionals most commonly listed patient non-adherence, unrealistic guideline goals and provider time-constraints. Providers thought that the guidelines included too much detail and identified the lack of a quick resource as a barrier to clinical implementation. Most were unaware of the Clinical Action Plans.

**Conclusions:**

Perceived barriers differed between renal clinicians and allied health professionals; educational and implementation tools tailored for different providers are needed.

## Background

Chronic kidney disease (CKD) represents a large burden among affected patients and on the healthcare system in the U.S. Approximately 26 million U.S. individuals have impaired kidney function or albuminuria [1]; the majority of these patients also have other significant co-morbid conditions such as diabetes mellitus, hypertension and cardiovascular disease [2]. Patients with CKD have significantly higher risk for hospital admissions [3-5], acute kidney injury [6], and cardiovascular-related death [7]. Moreover, CKD-associated costs have increased significantly, with CKD comprising 5.8% in 2000 to now 16% in 2009 of total Medicare costs. In 2009, this amounted to $33.8 billion in Medicare expenditures, excluding Medicare part D [2].

Studies have shown that early recognition and treatment of CKD can slow progression [8] to end-stage renal disease and improve overall survival among these patients [9]. In a cohort study of 556 patients initiating dialysis, receipt of quality care in the management of CKD-related complications prior to dialysis initiation was associated with greater survival during the first year following dialysis initiation [10]. To improve the recognition and facilitate management of patients with CKD, the National Kidney Foundation (NKF) developed the Kidney Disease Outcomes Quality Initiative (KDOQI). Since its inception in 1997, the KDOQI has provided clinicians with evidence and opinion-based practice guidelines covering pertinent clinical issues in CKD such as CKD staging, mineral bone disease and anemia among others. The KDOQI guidelines are supplemented by the Clinical Action Plans which are resources that yield management recommendations based on a patient’s stage of CKD and history of diabetes or hypertension. The Clinical Action Plans provide embedded relevant information from KDOQI guidelines. These guidelines and tools for clinicians are disseminated through the NKF’s website (http://www.kidney.org) and in print in special issues of the *American Journal of Kidney Diseases*.

While the KDOQI guidelines have been available and updated over the past decade, recognition and management of CKD patients remain highly variable and often suboptimal [11,12]. In a cross-sectional analysis of 198 patients with stage 4 and 5 CKD, only 10-17% of patients had achieved target blood pressures, 9-24% had intact parathyroid hormone (PTH) levels within recommended goal range, and 19-55% had optimal lipid levels based on recommended targets by the KDOQI guidelines [13]. Similar trends were observed in a large cohort of patients from the Southern California Kaiser Permanente organization from 2002 to 2008 [14]. To bridge the gap between clinical practice and guideline targets, the NKF KDOQI leadership formed an educational committee charged with identifying barriers to guideline utilization and implementation, determining clinician and patient educational needs, and development of effective tools to improve the care of patients with CKD. To that end, we conducted an online survey to evaluate renal providers’ familiarity, current use of and attitudes toward the KDOQI guidelines and NKF-provided clinical tools to implement KDOQI guidelines. The findings of this survey are to inform the KDOQI Education Committee regarding strategies to improve KDOQI guideline implementation.

## Methods

An online survey was developed by one of the authors (MJC), reviewed by members of the NKF KDOQI Education Committee, and offered from May to September 2010 to all physicians, physician extenders and allied health professionals, including nurses, dialysis technicians, dietitians and social workers who were registered in the NKF professional database. The survey was administered via Zoomerang (http://www.zoomerang.com) and included 38 multiple choice and open questions (Additional file 1). Providers were solicited twice and were not offered any compensation.

The survey asks about the respondent’s awareness, use and opinions regarding the presentation and content of KDOQI guidelines. An open-ended question asked about barriers to implementation of KDOQI guidelines in practice. Another set of questions focused on the surveyed providers awareness, use and opinion on presentation and content of specific guideline educational tools such as the Clinical Action Plans and the KDOQI information located within the NKF website. An open-ended question asked for suggested tools that would enhance guideline implementation. Respondent demographic information was also collected. All responses were compiled electronically by NKF staff. For questions regarding barriers to implementation of KDOQI guidelines in daily practice and what resources would make guidelines more useful for respondents’ practice, responses were assigned into one of 15 categories by two investigators independently. Responses which were categorized discordantly were then discussed to reach consensus. As the primary objective of this study was to query renal providers, data from responders who did not self-identify as nephrologists or renal allied health professionals were excluded. Renal providers were subsequently categorized into two groups: 1) clinicians including nephrologists, nurse practitioners and physician assistants; and 2) allied health professionals including registered nurses, social workers/case managers, dietitians/nutritionists, pharmacists and dialysis technicians. We also evaluated differences between nephrologists and nurse practitioners/physician assistants. Comparisons between the groups were performed using Fischer’s exact test and chi-square test, as appropriate. All statistical analyses were performed using Stata version 11.2 (StataCorp, College Station, TX).

## Results

The survey was sent to 16,323 providers. Of 951 health care providers who completed the survey, 71 resided outside of the U.S., 218 did not specify a subspecialty or did not respond to this question, and 59 self-identified as non-renal providers and were therefore excluded. Of the remaining 600 respondents who self-identified as renal healthcare providers, 160 were physicians, 49 were nurse practitioners/physician assistants, and 391 were allied health professionals, including registered nurses (n = 167), dietitians (n = 176), and other professionals (n = 48). The demographic and practice characteristics of the renal healthcare providers are summarized in Table 1.

**Table 1:** Characteristics of renal survey participants

	**No. of respondents**	**Providers**	**Allied health professionals**
**Number of respondents**	600	209	391
**Profession, n (%)**		160 (77) Physicians	167 (43) Registered Nurses
		49 (24) Physician Extenders	176 (45) Dietitians
			48 (12) Others*
**No. of years in practice, n (%)**	424		
**<6 years**	85 (20)	43 (20)	42 (11)
**6 years or longer**	339 (70)	105 (50)	234 (60)
**Practice settings, n (%)**	596		
**Clinic**	40 (7)	19 (9)	21 (5)
**Dialysis unit**	276 (46)	21 (10)	255 (65)
**Hospital**	100 (17)	33 (16)	67 (17)
**Private practice**	57 (10)	55 (26)	2 (1)
**University**	67 (11)	58 (28)	9 (2)
**Other**	56 (9)	21 (10)	35 (9)
**No. of patients seen per week, n (%)**	578		
**0-50**	214 (37)	73 (36)	141 (38)
**51-100**	193 (33)	72 (35)	121 (32)
**101-150**	120 (21)	40 (19)	80 (22)
**>150**	51 (9)	20 (10)	31 (8)

* Others include social workers (n = 17), dialysis technicians (n = 11), pharmacists (n = 8), and participants who responded “Other” for their profession (n = 12).

**Figure 1:**
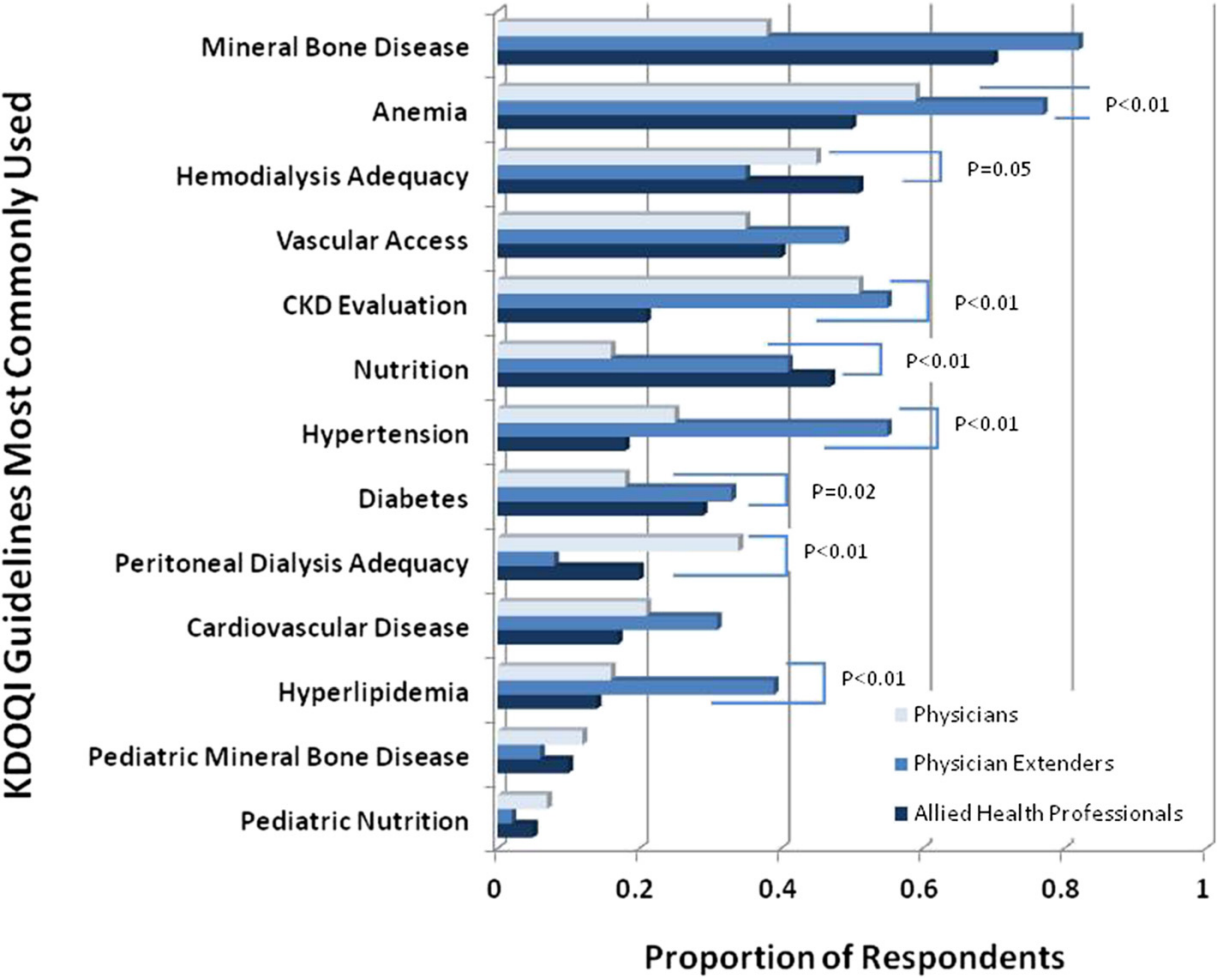
**KDOQI Guidelines Most Commonly Used by Renal Providers.** The proportions of respondents among physicians (light blue), physician extenders (medium blue) and allied health professionals (dark blue) for each KDOQI guideline topic are shown. P-values are detailed for topics in which there were significant differences in the proportion of respondents between the two groups.

Table 2 displays responses regarding use of the KDOQI guidelines stratified by renal providers. While 78% of all providers reported using the KDOQI guidelines in their practice, the proportion of physicians and physician extenders reporting often using these guidelines was lower compared to allied health professionals (69% vs. 83%, respectively; P < 0.001). Compared with physician extenders, fewer physicians reported using the KDOQI guidelines often (92% vs. 62%, respectively; p < 0.01) and at least once a month (65% vs, 41%, respectively; P = 0.01). Among nephrologists, the three KDOQI guidelines most commonly used within three months of the survey were anemia (59%), CKD evaluation (51%), and hemodialysis adequacy (45%). Among physician extenders, the top three KDOQI guidelines used within three months of the survey were mineral bone disease (82%), anemia (77%), and CKD evaluation/ hypertension (55%) (Figure 1). In contrast, the three KDQOI guidelines most commonly used in the three months prior to the survey among allied health professionals were mineral bone disease (70%), hemodialysis adequacy (51%), and nutrition (47%). Compared with clinicians, allied health professionals were more likely to have used the KDOQI guidelines pertaining to hemodialysis adequacy and nutrition and less likely to have used the KDOQI guidelines regarding anemia, CKD evaluation, hypertension, peritoneal dialysis, and hyperlipidemia within the 3 months prior to completing the survey.

**Table 2:** Responses to questions inquiring about KDOQI use by renal health care providers

**Questions**	**No. of respondents (%)**	**Physicians N (%)**	**Allied health professionals N (%)**	**P-value**
**Do you use the KDOQI guidelines in your practice?**	598			
**Often**	466 (78)	144 (69)	322 (83)	
**Sometimes**	107 (18)	51 (24)	56 (14)	
**Rarely or never**	25 (4)	14 (7)	11 (3)	<0.001
**How often do you look up a specific KDOQI guideline topic or recommendation?**	597			
**At least once a month**	281 (47)	99 (47)	182 (47)	
**Less than once a month or never**	316 (53)	110 (53)	206 (53)	0.82
**The guidelines are presented with the correct amount of information**	593			
**Too much detail**	129 (22)	56 (27)	73 (19)	
**Just right**	384 (65)	120 (58)	264 (68)	
**Too little**	80 (13)	30 (15)	50 (13)	0.04
**I find the KDQOI guidelines can be easily adapted to my practice**	596			
**Agree**	412 (69)	142 (68)	270 (70)	
**Neutral**	132 (22)	43 (21)	89 (23)	
**Disagree**	52 (9)	24 (11)	28 (7)	0.20

KDOQI, Kidney Disease and Outcomes Quality Initiative.

Of the renal providers, 67% thought that the KDOQI guidelines were presented with the correct amount of information. A similar proportion of nephrologists and physician extenders felt that the guidelines provided too much detail (15%). A greater proportion of these clinicians, however, reported that the guidelines provided too much detail compared with allied health professionals (27% vs. 19%, respectively; P = 0.04). However, renal providers felt that the KDOQI guidelines could be easily adapted to their clinical practice (69%).

Among the fourteen clinicians who reported rarely or never having used the KDOQI guidelines, 28% felt that they had insufficient time to read the guidelines or summary information, 21% could not find information they needed quickly, 14% practiced in settings with their own algorithms and guidelines, and 64% did not agree with the KDOQI guidelines. Among the eleven allied health professionals who reported rarely or never having used the KDOQI guidelines, 54% were not familiar with the KDOQI guidelines and felt that they did not have sufficient time to read the guidelines. In addition, 18% reported being unable to find needed information quickly within the guidelines and reported having algorithms and guidelines within their own practice or unit; only 9% did not agree with the KDOQI guidelines.

**Figure 2:**
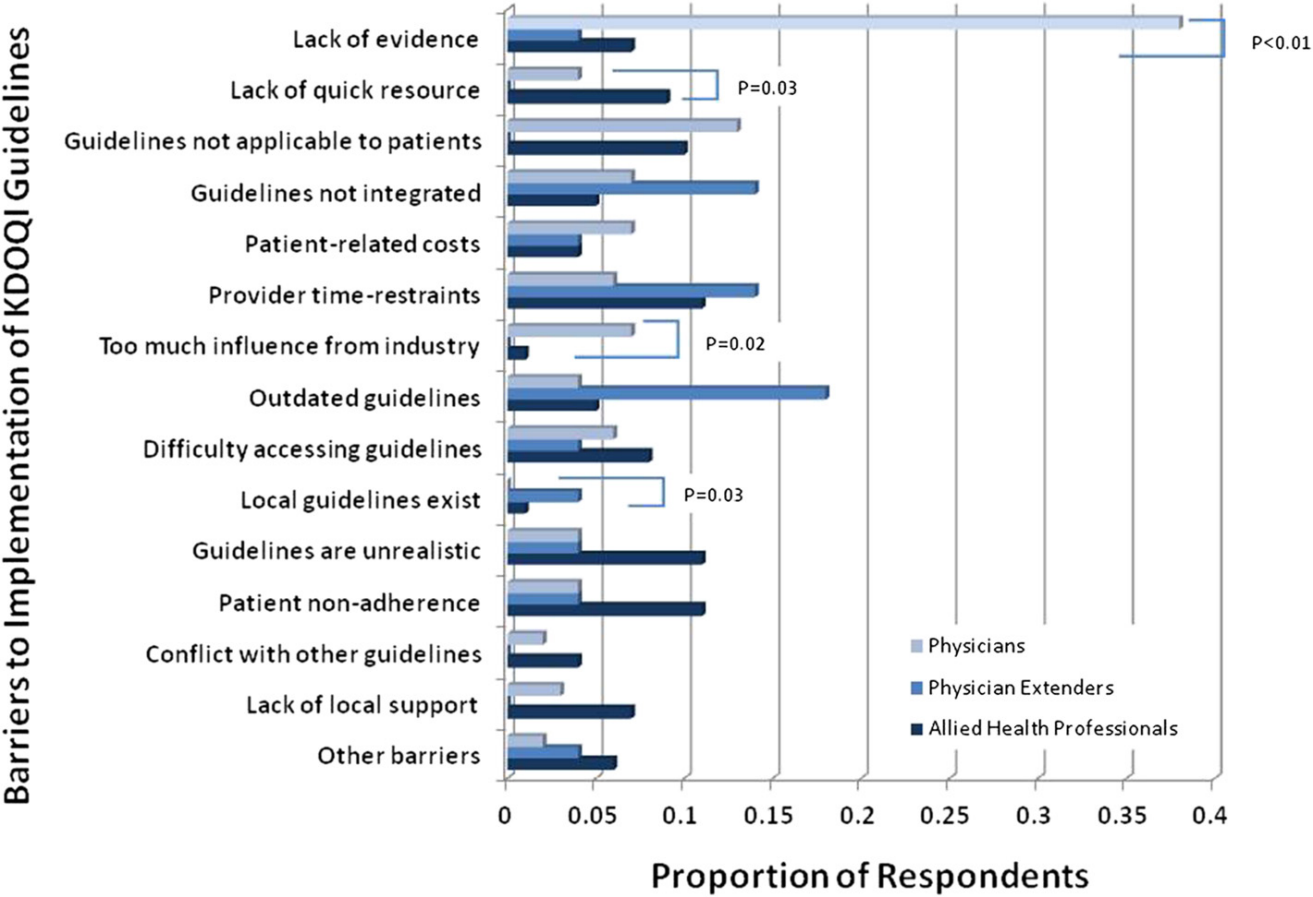
**Barriers to the Implementation of KDOQI Guidelines.** The proportions of respondents among physicians (light blue), physician extenders (medium blue) and allied health professionals (dark blue) for each barrier are shown. P-values are detailed for barriers in which there were significant differences in the proportion of respondents between the two groups.

When participants were asked to list barriers, if any, to the implementation of KDOQI guidelines to clinical practice, 46% of clinicians and 56% of allied health professionals identified no barriers (P = 0.03). Among barriers listed, the most common barrier perceived by physicians was the lack of evidence supporting the KDOQI guidelines (38%), followed distantly by the guidelines not being applicable to their patients (13%) (Figure 2). Compared to physician extenders and allied health professionals, a larger proportion of nephrologists cited lack of evidence and too much influence from industry as barriers to the implementation of the KDOQI guidelines. Among physician extenders, the most common barriers perceived were outdated guidelines (18%), lack of integration of the guidelines into their practice (14%), and provider time-constraints (14%). Among allied health professionals, the most common perceived barriers were patient non-adherence, unrealistic guidelines and provider time-constraints. Compared with physicians and physician extenders, a greater proportion of allied health professionals listed the lack of a quick resource as barriers to clinical implementation of the KDOQI guidelines.

To assess renal providers’ perceptions and use of the NKF’s Online Clinical Action Plans to facilitate translation of KDOQI guidelines into practice, respondents were queried regarding these tools, and the results are shown in Table 3. The majority of renal healthcare providers were not aware of the Clinical Action Plans, with a greater proportion of allied health professionals unaware of these tools compared to physicians and physician extenders (78% vs. 71%, respectively; P = 0.05). Moreover, the majority of renal healthcare providers reported rarely or never having used the Clinical Actions Plans (79%). Compared with physician extenders, physicians were slightly more likely to have rarely or never used the Clinical Action Plans (80% vs. 89%, respective; P = 0.01) but were otherwise similar in their response to the other questions regarding the Clinical Action Plans. Among the minority of respondents who had previously utilized the Clinical Action Plans, approximately one-half reported that they were easy to access, and 76% were neutral when asked whether the Clinical Action Plans were presented with the correct amount of information.

**Table 3:** Responses to questions inquiring about the NKF clinical action plans

**Questions**	**No. of respondents (%)**	**Providers N (%)**	**Allied health professionals N (%)**	**P-value**
**Are you aware of NKF’s online Clinical Action Plans?**	595			
**No**	449 (75)	148 (71)	301 (78)	
**Yes**	146 (25)	61 (29)	85 (22)	0.05
**Have you used the Clinical Actions Plans on the NKF Website?**	542			
**Often**	14 (3)	7 (4)	7 (2)	
**Sometimes**	51 (9)	18 (10)	33 (9)	
**Rarely or never**	477 (88)	160 (86)	317 (89)	0.43
**If you use them, the Clinical Action Plans are easy to access.**	128			
**Agree**	64 (50)	25 (51)	39 (49)	
**Neutral**	56 (44)	18 (37)	38 (48)	
**Disagree**	8 (6)	6 (12)	2 (3)	0.07
**If you use them, the Clinical Action Plans are presented with the correct amount of information.**	121			
**Agree**	25 (21)	13 (29)	12 (16)	
**Neutral**	92 (76)	31 (69)	61 (80)	
**Disagree**	4 (3)	1 (2)	3 (4)	0.21

NKF, National Kidney Foundation; KDOQI, Kidney Disease and Outcomes Quality Initiative.

**Figure 3:**
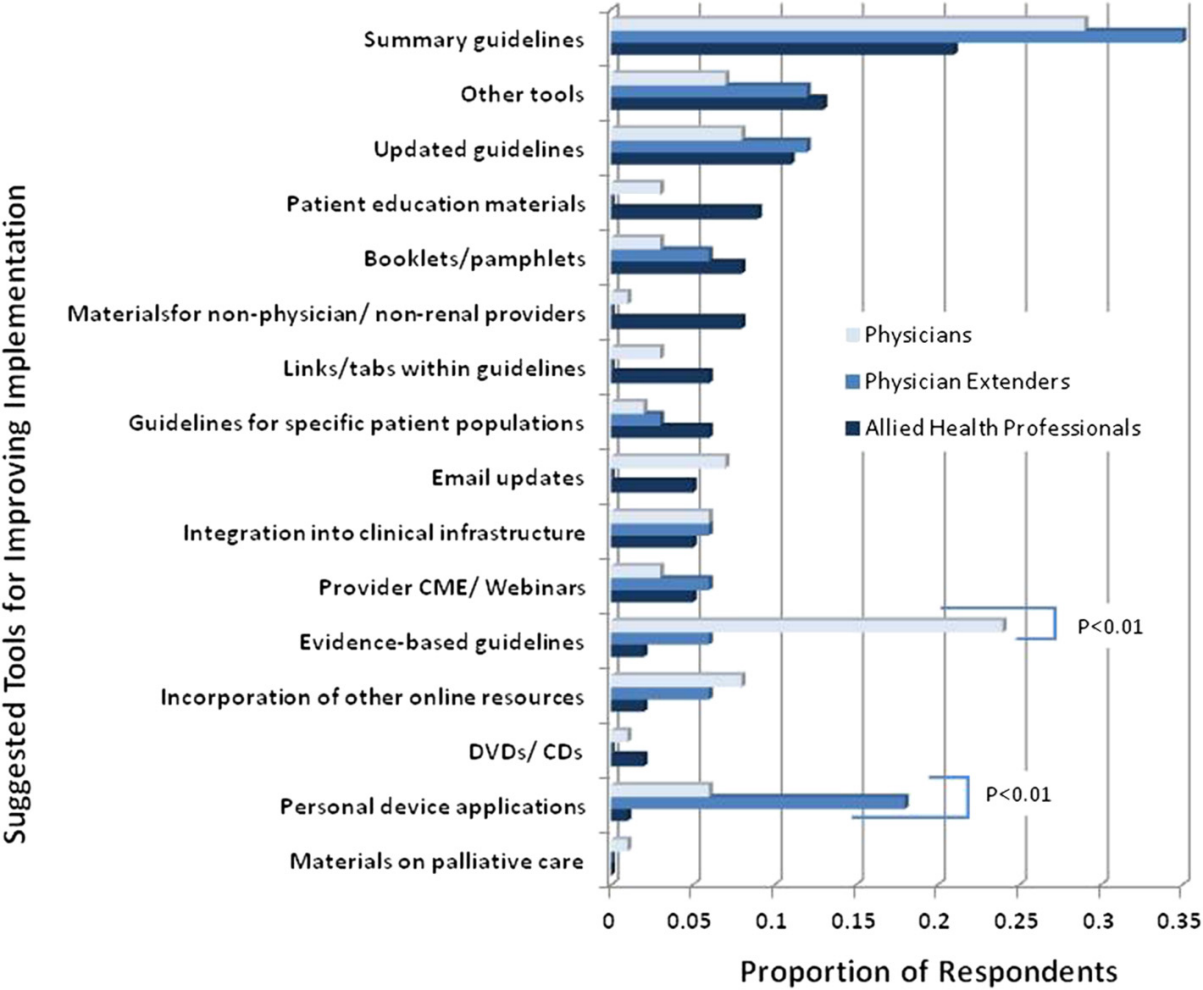
**Suggested Tools for Improving the Implementation of KDOQI Guidelines.** The proportions of respondents among physicians (light blue), physician extenders (medium blue) and allied health professionals (dark blue) for each suggested tool are shown. P-values are detailed for suggested tools in which there were significant differences in the proportion of respondents between the two groups.

Participants were also asked to list tools that they thought would facilitate the implementation of KDOQI guidelines into clinical practice. Among 218 renal providers who responded to this question, the most common tool suggested by providers was summary guidelines (Figure 3). A greater proportion of physicians, compared with physician extenders and allied health professionals, listed evidence-based guidelines (26% vs. 6% and 2%, respectively; P < 0.01) as a potential tool to improve implementation of KDOQI guidelines into clinical practice. In contrast, a larger proportion of physician extenders suggested personal device applications as potential tools for guideline implementation compared to physicians and allied health professionals (18% vs. 6% and 1%, respectively; P < 0.01). In addition, fewer physicians and physician extenders compared with allied health professionals suggested materials geared for non-physician providers (1% vs. 8%, respectively; P = 0.03) and patient education materials (2% vs. 9%, respectively; P = 0.05) as potential tools.

## Discussion

This report is the first to evaluate diverse nephrology health care providers’ current use and perceptions of the NKF KDOQI guidelines which have assumed the role of primary national documents and informs the “standard of care” for individuals with CKD in the U.S. Such knowledge will help inform the NKF KDOQI Educational Committee and other guiding institutions in nephrology determine renal providers’ current needs in order to provide the necessary knowledge to improve outcomes in patients with CKD. In this survey, we found that the majority of renal providers reported using the KDOQI guidelines often and felt that the KDOQI guidelines could be easily implemented into clinical practice. However, approximately one-half of respondents identified at least one translational barrier.

Several studies evaluating the use of clinical practice guidelines have suggested that provider adoption of guidelines in clinical practice is relatively low, particularly among physicians [15-17]. In these studies, low adherence to practice guidelines was often attributed to various barriers, including factors related to the guidelines themselves and to provider and patient perceptions and behaviors [15]. When obstacles to clinical implementation were identified among responders of our survey, physicians, in particular, most frequently cited the lack of evidence supporting KDOQI guidelines while allied health professionals most commonly listed patient non-adherence, unrealistic goals and provider time-constraints. Most of these barriers were previously described in the systematic review of barriers to physician adherence to clinical practice guidelines by Cabana and colleagues [15]. In this review, lack of physician awareness, familiarity and agreement with specific guidelines were among the physician-related barriers contributing to non-adherence with clinical practice guidelines. While most renal providers in our study were aware of KDOQI guidelines, the great majority were not aware of one the NKF’s tools to implement guidelines, the Clinical Action Plans. Moreover, the lack of agreement with specific guidelines appears to be relevant among renal physicians and physician extenders and may be related to the fact that the older KDQOI guidelines are largely based on observational data. Indeed, Strippoli and colleagues have poignantly demonstrated that the field of nephrology had the lowest number of randomized clinical trials between 1966 and 2002 among all other internal medicine subspecialties.[18] Furthermore, the quality of trial reporting in nephrology was poor with a significant number of studies lacking sufficient information on the masking process and failing to conduct “intention-to-treat analysis.” These observations are important as studies in other fields show that adherence to guidelines is greater when they are supported by evidence from randomized clinical trials [16]. This has recently been demonstrated within nephrology by the evolution of anemia management in patients with CKD based on recent randomized clinical trials of erythropoiesis-stimulating agents [19-21], emphasizing the importance of well-conducted trials in informing national guidelines and influencing provider perceptions of their credibility.

An additional potential barrier to the adoption of clinical practice guidelines previously reported among physicians is related to the guidelines themselves, with at least 10% of participants stating that guidelines are not easy to use, cumbersome, and confusing [15]. In our survey, a notable proportion of renal healthcare providers thought that the KDOQI guidelines included too much detail, and respondents cited the lack of a quick resource for the guidelines as a barrier to the clinical implementation of KDOQI guidelines. This may also be related to provider time-constraints which were also cited as a barrier by survey respondents. To address these particular barriers, several tools were suggested by survey respondents, with guideline summaries being the most common tool suggested. The NKF has also provided the Clinical Action Plans on its website to facilitate implementation of the KDOQI guidelines; unfortunately, most renal healthcare providers surveyed were unaware of these online tools. Moreover, of those who had previously used the Clinical Action Plans, only half reported that they were easy to access. This suggests that approaches to improve awareness and utilization of available clinical implementation tools among providers are needed. In addition, studies to determine the efficacy of such tools are needed.

While clinical practice guidelines assimilate the large volume of new scientific information into specific, clinically meaningful recommendations and to ultimately improve quality of care for patients with CKD [22], several obstacles hinder the application of these guidelines into clinical practice. Several endeavors to address these barriers and improve integration of guidelines into practice have been developed, and more are underway. The most notable endeavor has been the automatic reporting of estimated glomerular filtration rates (eGFR); however, early assessments of this initiative have underscored the need for provider education. While eGFR reporting has led to improved recognition of patients with CKD and increased numbers of nephrology referrals [23], a chart review of nearly 15,000 primary care patients in the Bronx Veterans Affairs Medical Center showed that eGFR reporting alone had no significant effect on the actual care of patients with stage 3 CKD [24]. While tools geared for educating renal providers such as those suggested by survey respondents play a role in helping implement guidelines, other strategies are required. Clinical decision support systems have been endorsed by the Institute of Medicine to improve implementation of guidelines [25]. Studies within the Veterans Affairs using clinical decision support systems that present clinical reminders to healthcare providers about general appropriate management of chronic conditions such as hypertension and diabetes have shown improved adherence with guidelines [26]. We have shown that there are variations in KDOQI topics most often used by specific providers and in the perceived barriers between various renal providers in our study. Therefore, it is likely that such systems will need tailoring based on the type of provider and his/her role in the care of patients with CKD as well as the provider work setting. Matchar and colleagues conducted a multidisciplinary needs assessment to develop a conglomerate of tools that could aid in the identification of patients with CKD, development and implementation of tailored management plans [27]. These were based on four clinical practice contexts: 1) primary care providers with a hectic practice but no significant knowledge of CKD issues; 2) primary care providers practicing in a rural area with limited access to nephrology consultation; 3) renal physicians overwhelmed with patients but with good rapport with referring providers; and 4) renal physicians who have suboptimal communication with primary providers and primarily received referrals for very advanced CKD. The investigators’ efforts in collaboration with the Renal Physicians Association and the National Institutes of Health National Kidney Disease Education Program culminated in the *Advanced CKD Patient Management Toolkit* (http://www.renalmd.org/RPA-Advanced-CKD-Patient-Management-Toolkit) which was pilot-tested in two nephrology clinical practices in 2005, further refined and tested in 10 nephrology clinical practices, and was most recently revised in 2009 to help providers caring for patients with CKD, particularly nephrologists, implement clinical practice guidelines into their practice [28]. While geared for nephrologists, the tookit includes several assessment and implementation tools that may be useful for primary care providers and patients as well. In the United Kingdom, CKD-focused clinical decision support systems and electronic patient tools have been developed and put into practice [29]. Prospective studies are needed, however, to determine whether these efforts translate into improved outcomes in patients with CKD.

The findings reported in our paper should be interpreted in the context of its limitations. A major limitation is the low overall response rate of 5.8%. Although this response rate may be due to technical issues with survey delivery, such as invalid email addresses, we cannot rule out other factors related to non-response that may have biased our findings. In addition, we were unable to determine the response rate among U.S. renal providers specifically as data on the profession of non-responders were unknown. Thirdly, the online survey was sent only to users of the NKF website. Due to the focused data obtained through the survey and the limited data available on U.S. renal providers, we were unable to determine whether survey responders were comparable to non-responders. Therefore, self-reported practices and attitudes toward the KDOQI guidelines and tools may not be generalizable to all U.S. renal healthcare providers. These survey results, however, included responses from various types of providers involved in the care of patients with CKD and sheds light on some aspects of the KDOQI guidelines and related tools that could help professional associations improve delivery of educational and implementation tools to specific subgroups of renal providers. We excluded individuals who self-identified as having subspecialties other than nephrology; therefore, a thorough assessment of the utilization and opinions of the KDOQI guidelines of other subspecialties, especially primary care providers who bear the burden of CKD care, are still needed in order to assess the educational needs of these clinicians.

## Conclusions

In summary, the KDOQI guidelines are commonly used by renal healthcare providers; however, barriers to the implementation of KDOQI guidelines into clinical practice exist. The topics of interest and the perceived barriers differ among physicians, physician extenders and allied health professionals; therefore, educational and implementation tools tailored for different providers are needed.

## Competing interests

The authors declare that they have no competing interests.

## Authors’ contributions

MME participated in drafting the manuscript and performing statistical analyses. BGJ and MJC participated in designing the study and helped to draft the paper. CHF, SSS, EH, MVR and MAP were involved in editing drafts of the manuscript. All authors read and approved the manuscript.

## Authors’ information

M.J.C. serves as the KDOQI Vice-Chair of Education for the National Kidney Foundation.

## Pre-publication history

The pre-publication history for this paper can be accessed here:

http://www.biomedcentral.com/1471-2369/14/230/prepub

## Supplementary Material

Additional file 1 NKF/KDOQI Education Survey. Click here for file

## Support

M.M.E. is supported by NIH-NIDDK grant K23DK081317. K.L.C. is supported by NIH-NIDDK K23DK080952 and an American Society of Nephrology Carl W. Gottschalk Research Scholar Grant.

## References

[B1] Coresh J Selvin E Stevens LA Manzi J Kusek JW Eggers P Van Lente F Levey AS Prevalence of chronic kidney disease in the United States JAMA 2007 14 17 2038 2047 17986697 10.1001/jama.298.17.2038

[B2] U.S. Renal Data System, USRDS 2011 Annual Data Report Atlas of Chronic Kidney Disease and End-Stage Renal Disease in the United States 2010 Bethesda, MD: National Institutes of Health, National Institute of Diabetes and Digestive and Kidney Diseases In

[B3] Go AS Yang J Ackerson LM Lepper K Robbins S Massie BM Shlipak MG Hemoglobin level, chronic kidney disease, and the risks of death and hospitalization in adults with chronic heart failure: the Anemia in Chronic Heart Failure: Outcomes and Resource Utilization (ANCHOR) Study Circulation 2006 14 23 2713 2723 16754803 10.1161/CIRCULATIONAHA.105.577577

[B4] James MT Quan H Tonelli M Manns BJ Faris P Laupland KB Hemmelgarn BR CKD and risk of hospitalization and death with pneumonia Am J Kidney Dis 2009 14 1 24 32 19447535 10.1053/j.ajkd.2009.04.005

[B5] Nitsch D Nonyane BA Smeeth L Bulpitt CJ Roderick PJ Fletcher A CKD and hospitalization in the elderly: a community-based cohort study in the United Kingdom Am J Kidney Dis 2011 14 5 664 672 21146270 10.1053/j.ajkd.2010.09.026 PMC3392651

[B6] Grams ME Astor BC Bash LD Matsushita K Wang Y Coresh J Albuminuria and estimated glomerular filtration rate independently associate with acute kidney injury J Am Soc Nephrol 2010 14 10 1757 1764 20671214 10.1681/ASN.2010010128 PMC3013549

[B7] Matsushita K van der Velde M Astor BC Woodward M Levey AS de Jong PE Coresh J Gansevoort RT Association of estimated glomerular filtration rate and albuminuria with all-cause and cardiovascular mortality in general population cohorts: a collaborative meta-analysis Lancet 2010 14 9731 2073 2081 20483451 10.1016/S0140-6736(10)60674-5 PMC3993088

[B8] Jones C Roderick P Harris S Rogerson M Decline in kidney function before and after nephrology referral and the effect on survival in moderate to advanced chronic kidney disease Nephrol Dial Transplant 2006 14 8 2133 2143 16644779 10.1093/ndt/gfl198

[B9] Stack AG Impact of timing of nephrology referral and pre-ESRD care on mortality risk among new ESRD patients in the United States Am J Kidney Dis 2003 14 2 310 318 12552491 10.1053/ajkd.2003.50038

[B10] Thilly N Boini S Loos-Ayav C Kessler M Briancon S Frimat L Impact of predialysis therapeutic practices on patient outcomes during the first year of dialysis: the Pharmacoepidemiologic AVENIR study Med Care 2012 14 1 35 42 20808260 10.1097/MLR.0b013e3181d56926

[B11] McClellan WM Wasse H McClellan AC Kipp A Waller LA Rocco MV Treatment center and geographic variability in pre-ESRD care associate with increased mortality J Am Soc Nephrol 2009 14 5 1078 1085 19321704 10.1681/ASN.2008060624 PMC2678038

[B12] Charles RF Powe NR Jaar BG Troll MU Parekh RS Boulware LE Clinical testing patterns and cost implications of variation in the evaluation of CKD among US physicians Am J Kidney Dis 2009 14 2 227 237 19371991 10.1053/j.ajkd.2008.12.044 PMC2714476

[B13] Lenz O Mekala DP Patel DV Fornoni A Metz D Roth D Barriers to successful care for chronic kidney disease BMC Nephrol 2005 14 11 16250919 10.1186/1471-2369-6-11 PMC1283975

[B14] Rutkowski M Mann W Derose S Selevan D Pascual N Diesto J Crooks P Implementing KDOQI CKD definition and staging guidelines in Southern California Kaiser Permanente Am J Kidney Dis 2009 14 3 Suppl 3 S86 S99 19231766 10.1053/j.ajkd.2008.07.052

[B15] Cabana MD Rand CS Powe NR Wu AW Wilson MH Abboud PA Rubin HR Why don’t physicians follow clinical practice guidelines? A framework for improvement JAMA 1999 14 15 1458 1465 10535437 10.1001/jama.282.15.1458

[B16] Leape LL Weissman JS Schneider EC Piana RN Gatsonis C Epstein AM Adherence to practice guidelines: the role of specialty society guidelines Am Heart J 2003 14 1 19 26 12514650 10.1067/mhj.2003.35

[B17] Maue SK Segal R Kimberlin CL Lipowski EE Predicting physician guideline compliance: an assessment of motivators and perceived barriers Am J Manag Care 2004 14 6 383 391 15209482

[B18] Strippoli GF Craig JC Schena FP The number, quality, and coverage of randomized controlled trials in nephrology J Am Soc Nephrol 2004 14 2 411 419 14747388 10.1097/01.asn.0000100125.21491.46

[B19] Drueke TB Locatelli F Clyne N Eckardt KU Macdougall IC Tsakiris D Burger HU Scherhag A Normalization of hemoglobin level in patients with chronic kidney disease and anemia N Engl J Med 2006 14 20 2071 2084 17108342 10.1056/NEJMoa062276

[B20] Singh AK Szczech L Tang KL Barnhart H Sapp S Wolfson M Reddan D Correction of anemia with epoetin alfa in chronic kidney disease N Engl J Med 2006 14 20 2085 2098 17108343 10.1056/NEJMoa065485

[B21] Pfeffer MA Burdmann EA Chen CY Cooper ME de Zeeuw D Eckardt KU Feyzi JM Ivanovich P Kewalramani R Levey AS A trial of darbepoetin alfa in type 2 diabetes and chronic kidney disease N Engl J Med 2009 14 21 2019 2032 19880844 10.1056/NEJMoa0907845

[B22] Eknoyan G Why we need clinical practice guidelines in chronic kidney disease J Ren Nutr 2010 14 5 Suppl S127 S130 20797561 10.1053/j.jrn.2010.06.014

[B23] Jain AK McLeod I Huo C Cuerden MS Akbari A Tonelli M van Walraven C Quinn RR Hemmelgarn B Oliver MJ When laboratories report estimated glomerular filtration rates in addition to serum creatinines, nephrology consults increase Kidney Int 2009 14 3 318 323 19436331 10.1038/ki.2009.158

[B24] Wyatt C Konduri V Eng J Rohatgi R Reporting of estimated GFR in the primary care clinic Am J Kidney Dis 2007 14 5 634 641 17472845 10.1053/j.ajkd.2007.02.258

[B25] Patwardhan MB Kawamoto K Lobach D Patel UD Matchar DB Recommendations for a clinical decision support for the management of individuals with chronic kidney disease Clin J Am Soc Nephrol 2009 14 2 273 283 19176797 10.2215/CJN.02590508 PMC2637586

[B26] Jha AK Perlin JB Kizer KW Dudley RA Effect of the transformation of the Veterans Affairs Health Care System on the quality of care N Engl J Med 2003 14 22 2218 2227 12773650 10.1056/NEJMsa021899

[B27] Matchar DB Patwardhan MB Samsa GP Haley WE Facilitated process improvement: an approach to the seamless linkage between evidence and practice in CKD Am J Kidney Dis 2006 14 3 528 538 16490633 10.1053/j.ajkd.2005.11.016

[B28] How to utilize RPA’s Advanced CKD Patient Management Toolkit Renal Physicians Association 2012

[B29] Stevens PE de Lusignan S Farmer CK Tomson CR Engaging primary care in CKD initiatives: the UK experience Nephrol Dial Transplant 2012 14 Suppl 3 iii5 iii11 23115141 10.1093/ndt/gfs103

