# Supplementary material for: Perceptions and use of the national kidney foundation KDOQI guidelines: a survey of U.S. renal healthcare providers
Source: BMC Nephrol. 2013 Oct 24;14:230. doi: 10.1186/1471-2369-14-230 (PMC4016578; doi:10.1186/1471-2369-14-230)
Supplement: Additional file 1 — NKF/KDOQI Education Survey. [file 1471-2369-14-230-S1.docx]

**NKF/KDOQI EDUCATION SURVEY**

**KDOQI Guidelines The National Kidney Foundation Kidney Disease Outcome Quality Initiative (NKF KDOQI) ™ has published evidence-based clinical practice guidelines for all stages of chronic kidney disease (CKD) and related complications since 1997. To date, KDOQI has developed and disseminated thirteen guidelines for the care of kidney disease patients.**

**1. Do you use the KDOQI guidelines in your practice**

a) Often

b) Sometimes

c) Rarely

d) Never

**2. If you answered Rarely or Never, which of the following (check all) would apply?**

a) I’m not familiar with the KDOQI guidelines

b) Not enough time to read the guidelines or summary information

c) I can’t find the information I need quickly from the guidelines

d) My practice/ unit uses their own algorithms and guidelines

e) I don’t agree with the KDOQI guidelines

f) Other, please comment below

**3. Where do you collect your guideline information? (Please select all that apply)**

a) NKF Website

b) NKF Educational Materials

c) AJKD

d) Medscape/ Web MD

e) Conferences

f) Other, please specify

**4. How often do you look up a specific KDOQI guideline topic or recommendation?**

a) Once or more weekly

b) Once or more monthly

c) Less than once a month

d) Never

**5. Which KDOQI guidelines have you used in the past 3 months?**

-Diabetes in Chronic Kidney Disease

-Anemia in Chronic Kidney Disease

-Chronic Kidney Disease; Evaluation, Classification, and Stratification

-Bone Metabolism and Disease in Children with Chronic Kidney Disease

-Hypertension and Antihypertensive Agents in Chronic Kidney Disease

-Managing Dysplipidemia in Chronic Kidney Disease

-Nutrition in Children with CKD: 2008 Update

-Nutrition in Chronic Renal Failure

-Hemodialysis Adequacy

-Peritoneal Dialysis Adequacy

-Vascular Access

-Cardiovascular Disease in Dialysis Patients

**6. The KDOQI guidelines are presented with the correct amount of information:**

a) Too much detail

b) Right amount of detail

c) Too little detail

**7. I find the KDOQI Guidelines can be easily adapted to my practice:**

a) Strongly agree

b) Somewhat agree

c) Neutral

d) Somewhat disagree

e) Strongly disagree

**8. Please list one or more barriers making it difficult to apply to my day-to-day practice:**

**9. What resources or other means would make the guidelines more useful to your practice?**

**NKF Professional Educational Resources NKF educational materials, including, printed and online materials, are designed to support and enhance implementation of KDOQI and KDIGO guideline recommendations. NKF publishes materials for both patients and professionals. Relative to NKF PROFESSIONAL resources:**

**10. Are you aware of NKF’s professional educational materials**

a) Yes

b) No

**11. I use NKF educational resources (print or online) to help me apply the guidelines:**

a) Often

b) Sometimes

c) Rarely

d) Never, please explain

**12. Check one or several KDOQI Guidelines for which you would like to have NKF educational tools:**

-Diabetes in Chronic Kidney Disease

-Anemia in Chronic Kidney Disease

-Chronic Kidney Disease; Evaluation, Classification, and Stratification

-Bone Metabolism and Disease in Children with Chronic Kidney Disease

-Hypertension and Antihypertensive Agents in Chronic Kidney Disease

-Managing Dysplipidemia in Chronic Kidney Disease

-Nutrition in Children with CKD: 2008 Update

-Nutrition in Chronic Renal Failure

-Hemodialysis Adequacy

-Peritoneal Dialysis Adequacy

-Vascular Access

-Cardiovascular Disease in Dialysis Patients

**13. Check one or several KDOQI Commentaries of KDIGO Guidelines for which you would like to have NKF educational tools:**

-Hepatitis C

-Diagnosis, Evaluation, Prevention and Treatment of Chronic Kidney Disease Related Mineral and Bone Disorders (CKD-MBD)

-Care of the Kidney Transplant Recipients

**NKF Online Clinical Action Plans NKF’s Clinical Action Plans of evidence-based CKD interventions have been expanded into multidisciplinary patient care plans for nurses, dietitians, MDs, dialysis technicians and social workers and are available online.**

**14. Are you aware of NKF’s online Clinical Action Plans?**

a) Yes

b) No

**15. Have you used the Clinical Action Plans on the NKF website?**

a) Often

b) Sometimes

c) Rarely

d) Never, please explain

**16. If you use them, the Clinical Action Plans are easy to access:**

a) Strongly agree

b) Somewhat agree

c) Neutral

d) Somewhat disagree

e) Strongly disagree

**17. If you use them, the Clinical Action Plans are presented with the correct amount of information:**

a) Too much detail

b) Right amount of detail

c) Too little detail

**18. Please tell us how we can improve our Clinical Action Plans:**

**NKF CME/CE The National Kidney Foundation is accredited by the Accreditation Council for Continuing Medical Education (CME) to provide continuing medical education for physicians. NKF also is approved to provide CE credits for Nurses, Dietitians and Social Workers, and works with other organizations to provide credits in other disciplines.**

**19. Have you participated in a NKF CME/ CE activity?**

a) Yes

b) No

**20. Did you find the CME/ CE program useful for applying guidelines to your practice?**

a) Often

b) Sometimes

c) Rarely

d) Never

**21. What topics would interest you for future CME/ CE programs?**

**22. Rank the following in the order of preference for future CME/ CE programs?**

-Web

-Print

-Live

-Audio

-Video/ DVD

-Television

-Applications for hand held devices

-Other

**23. Please describe why prefer these methods:**

**KDOQI Website General Questions NKF has a unique website for KDOQI, www.kdoqi.org which can be accessed directly or through the NKF website.**

**24. Are you aware of the KDOQI website?**

a) Yes

b) No

**25. How often do you use the KDOQI website?**

a) Once or more weekly

b) Once or more monthly

c) Less than once a month

d) Never

**26. The general content of the KDOQI website is useful:**

a) Strongly agree

b) Somewhat agree

c) Neutral

d) Somewhat disagree

e) Strongly disagree

**27. The general content of the KDOQI website is current:**

a) Strongly agree

b) Somewhat agree

c) Neutral

d) Somewhat disagree

e) Strongly disagree

**28. The KDOQI website is easy to navigate:**

a) Strongly agree

b) Somewhat agree

c) Neutral

d) Somewhat disagree

e) Strongly disagree

**29. The KDOQI website is easily readable:**

a) Strongly agree

b) Somewhat agree

c) Neutral

d) Somewhat disagree

e) Strongly disagree

**30. I refer my patients to the NKF website:**

a) Often

b) Sometimes

c) Rarely

d) Never, please comment

**General Questions**

**31. Please indicate your profession**

a) Physician

b) Physician Assistant

c) Nurse Practitioner

d) Nurse

e) Social Worker

f) Dietitian

g) Pharmacist

h) Dialysis Technician

i) Other:

**32. My specialty would be best described as:**

a) Nephrology

b) Primary Care

c) Cardiology

d) Endocrinology/ Diabetes

e) Urology

f) Other:

**33. Where do you practice (please all that apply)?**

-Clinic

-Dialysis Unit

-Hospital

-Private practice

-University

-Other:

**34. Approximately how many patients do you see weekly?**

a) 0-50

b) 51-100

c) 101-150

d) 151-200

e) More than 200

**35. How many years have you been in practice?**

a) 0-5

b) 6-10

c) 10-15

d) 15-20

e) 20-25

f)>30

**36. If you practice in the United States, what is your zip code?**

**37. If you practice outside the United States, in what country?**

**38. Please write final comments here:**
